# Novel application of the matched case–control design to compare food supply chains during an *Escherichia coli* O157 outbreak, United Kingdom, 2016

**DOI:** 10.2807/1560-7917.ES.2018.23.18.17-00195

**Published:** 2018-05-03

**Authors:** Thomas Inns, Paul Cleary, Nick Bundle, Sarah Foulkes, Ashley Sharp, Lara Utsi, Chris McBrien, Rehman Teagle, Alison Waldram, Chris Williams, Cathy McCann, Rob Smith, Sepeedeh Saleh, Noel McCarthy, Roberto Vivancos, Jeremy Hawker, Valerie Decraene

**Affiliations:** 1 Field Epidemiology Service, Public Health England, London, United Kingdom; 2 Institute of Psychology, Health and Society, University of Liverpool, Liverpool, United Kingdom; 3 NIHR Health Protection Research Unit in Gastrointestinal Infections, University of Liverpool, Liverpool, United Kingdom; 4 Field Epidemiology Training Programme, Public Health England, London, United Kingdom; 5 North West Health Protection Team, Public Health England, Liverpool, United Kingdom; 6 Communicable Disease Surveillance Centre, Public Health Wales, Cardiff, United Kingdom; 7 Warwick Medical School, University of Warwick, Warwick, United Kingdom; 8 NIHR Health Protection Research Unit in Emerging and Zoonotic Infections, University of Liverpool, Liverpool, United Kingdom

**Keywords:** Escherichia coli O157, Epidemiology, food-borne infections, gastrointestinal disease

## Abstract

There is a need for innovative methods to investigate outbreaks of food-borne infection linked to produce with a complex distribution network. The investigation of a large outbreak of *Escherichia coli* O157 PT34 infection in the United Kingdom in 2016 indicated that catering venues associated with multiple cases had used salad leaves sourced from one supplier. Our aim was to investigate whether catering venues linked to cases were more likely to have used salad leaves from this supplier. We conducted a matched case–control study, with catering venues as the units of analysis. We compared venues linked to cases to those without known linked cases. We included 43 study pairs and obtained information on salad leaf products received by each venue. The odds of a case venue being supplied with salad leaves by Supplier A were 7.67 times (95% confidence interval: 2.30–25.53) those of control venues. This association provided statistical evidence to support the findings of the other epidemiological investigations undertaken for this outbreak. This is a novel approach which is labour-intensive but which addresses the challenge of investigating exposures to food across a complex distribution network.

## Introduction

Shiga toxin-producing *Escherichia coli*, such as *E. coli* O157 have the potential to cause severe gastrointestinal disease, with infection leading to haemolytic uraemic syndrome (HUS) in 5–14% of cases [1]. The incidence of *E. coli* O157 has increased in England and Wales since 2005; incidence is highest in children younger than 10 years [2]. There have been a number of large *E. coli* O157 outbreaks in the United Kingdom (UK) [3,4], several linked to contaminated foods with wide distribution networks [5-7]. Food traceback investigations can be important for determining the source of *E. coli* outbreaks caused by contaminated food items [8-10].

A large outbreak of *E. coli* O157 PT34 was reported in the UK in 2016; an overview of the outbreak and associated investigations is published in this issue [11]. Briefly, an initial cluster of cases was detected in June 2016. Case–control and case–case studies were undertaken early in the outbreak investigation, which identified that consumption of mixed salad leaves and eating out at catering venues were significantly associated with illness [11]. Initial information from food chain investigations suggested that a number of catering venues associated with multiple cases had used salad leaves that were ultimately sourced from one company: Supplier A.

We undertook this study to investigate whether catering venues linked to cases were more likely to have used salad leaves from Supplier A than venues with no known links to cases.

## Methods

### Case definition

We defined a case as a person resident in the UK or the Channel Islands with a reference laboratory-confirmed infection of *E. coli* O157:H7 PT34 *eae + stx2 + stx1-*, within the outbreak single nucleotide polymorphism (SNP) cluster. We excluded cases if they were under 18 years-old, had a history of foreign travel or close contact with other individuals with gastroenteritis in the 10 days before onset, were a secondary case of a confirmed *E. coli* O157 infection or were asymptomatic.

### Venue definition and selection

We defined case venues as any catering venue which served salad leaves to one or more cases in the 10 days before their illness onset. We identified case venues from routine information gathered from cases through telephone or face-to-face interview. We defined control venues as any venue serving salad leaves and not known to be associated with any cases. Five classifications of venue were used: restaurant, bar, pub, café and takeaway (including sandwich shops). Case venues which did not fall into one of these classifications were excluded as it was not feasible to identify comparable control venues. We used Google Maps to identify, for each case venue, the three nearest food venues of the same classification, and applied the following exclusion criteria to both case and control venues: part of a national chain or franchise, or did not serve salad leaves (ascertained from online menus).

We applied a matched case–control design, with catering venues as the units of analysis. One control venue was included for each case venue. Reported exposure to ‘salad’ or ‘side salad’ was assumed to contain salad leaves. The food venue geographically closest to each case venue that was not known to be associated with outbreak cases and did not meet any of the exclusion criteria was included in the study. Where on further enquiry a venue was found to no longer be trading, we contacted the next closest venue which met the selection criteria.

### Data collection

Environmental Health Officers (EHOs) from local authorities undertook data collection for each venue following a standard protocol. EHOs asked study venues to provide information on the type, quantity, delivery dates and supplier details of salad leaf products received in the study period (1 to 30 June 2016). EHOs continued traceback investigation of the supply chain until the producer or importer was identified. Data were collected from 11 to 29 July 2016 and entered into Excel files in the format used by FoodChain-Lab [12].

### Data analysis

We tested the hypothesis that case venues were more likely than control venues to have been supplied by leaves from Supplier A. The exposure was defined as having received salad leaves from Supplier A in the study period. The analysis was performed using odds ratios (OR) and simple conditional logistic regression. Data analysis was carried out using Stata 13. We conducted sensitivity analyses to examine the effect of different study inclusion criteria.

### Survey of resource use

Given the novel nature of this study, we also captured information on the resources used. To do this, we asked public health staff and EHOs involved in the study to complete an online questionnaire to record staff time spent on the study, approximate salaries of respondents and feedback on the materials and process. These questionnaires were completed between 3 and 17 August 2016. We undertook a descriptive analysis using Stata 13.

## Results

### Case–control study

We identified 43 case venues and 43 control venues. Data were obtained for all 86 study venues. The 43 case venues were associated with 57 cases. Supplier A supplied 30 case venues and 10 control venues with salad leaves (Figure). One venue was linked to eight cases, one venue was linked to three cases, five venues were linked to two cases and 36 venues were linked to one case. The 30 case venues supplied by Supplier A were associated with 44 cases; the 13 case venues not known to have been supplied by Supplier A were associated with 13 cases.

**Figure:**
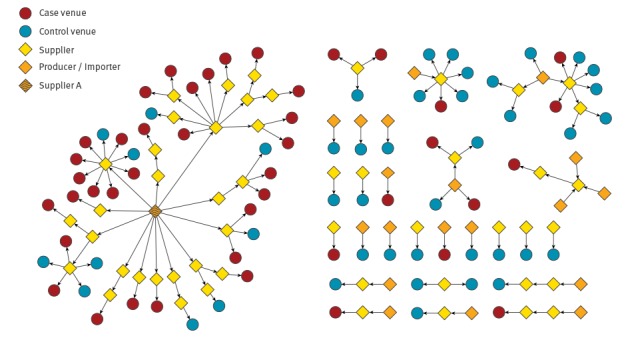
Food chain of salad leaves supplied to study venues, *Escherichia coli* O157 outbreak, United Kingdom, June 2016 (n = 86)

The case and control venue pairs are shown in Table. The most common venue types were cafés (n = 17), pubs (n = 13) and restaurants (n = 10). The mean distance between case and control pairs was 1.43 km (range: 0.0–5.75). The analysis indicated that the odds of a venue being supplied with salad leaves by Supplier A were 7.7 (95% confidence interval (CI): 2.3–25.5) times higher for case venues, compared with control venues. We conducted a sensitivity analysis which excluded venue pairs where the case had also eaten at another case venue linked to Supplier A. The association was greater in this subset (OR = 12; 95% CI: 2.8–48.8). In a sensitivity analysis including only those case venues linked to more than one case (n = 7), it was not possible to estimate an association.

**Table:** Description of matched case–control pairs with exposure status defined as being supplied with salad leaves by Supplier A, *Escherichia coli* O157 outbreak, United Kingdom, June 2016 (n = 43)

		Case exposed/ control exposed	Case exposed/ control unexposed	Case unexposed/ control exposed	Case unexposed/ control unexposed	Total
**Number of pairs**		**7**	**23**	**3**	**10**	**43**
Venue type	Restaurant	2	6	0	2	10
	Bar	0	1	0	0	1
	Pub	1	6	1	5	13
	Café	4	10	2	1	17
	Takeaway	0	0	0	2	2
**Mean pair distance (km)**		**1.13**	**1.37**	**0.54**	**2.03**	**1.43**
Pairs where the case had eaten at another case venue linked to Supplier A		0	0	1	2	3
Pairs where more than one case ate at the case venue		2	5	0	0	7

### Study resource survey

The survey of study resources was completed by 38 of 43 persons involved in the study: 12 Public Health England (PHE)/Public Health Wales (PHW) staff and 26 EHOs from local authorities involved in the investigation. These respondents estimated that they spent a total of 527 h on the study: 366 h for PHE/PHW staff and 161 h for EHOs. Of the PHE/PHW staff time, 148 h were spent on coordination of the study and liaison with data collectors. Using an approximation of staff gross hourly pay to indicate seniority of staff involved, we estimated that the total staff cost of these 527 hours was GBP 9,123 (EUR 10,470).

## Discussion

When investigating community outbreaks linked to contaminated foodstuffs, it may be possible to identify a common supplier between a subset of cases, but it may not be clear whether this apparent association has arisen by chance. We present a methodology that investigators can use to answer this question. We found that venues which served salad leaves to cases were significantly more likely to have been supplied with salad leaves from Supplier A than matched venues which were not known to have served salad leaves to cases. This association provided statistical evidence to support the findings of the other epidemiological investigations undertaken for this outbreak [11].

Despite extensive sampling of salad leaves from Supplier A, there were no positive microbiological findings to support the epidemiological and food chain evidence [11]. The absence of microbiological evidence is common in dispersed outbreaks linked to products with a short shelf life [13-15]. In such outbreaks, an analytical rather than descriptive approach to food chain investigations can provide stronger evidence for public health action. One method is to produce a ‘tracing score’ based on food chain networks and batch numbers [12]; another is to compare the food chain network to the phylogeny of cases (if genetic information is available) [16].

The study design applied here is a novel approach which addressed the challenge of investigating food exposures distributed widely through the food chain to a variety of catering venues. We have shown that this study design is feasible. The advantage of this approach over other analytical food chain study designs is that it does not require batch numbers or genetic information, that it provides a quantitative output which measures the strength of the association and that it is widely understood by those working in public health. The evidence provided by this study was important for the decision-making of the outbreak control team and would have been difficult to obtain in any other way.

There are potential alternatives to the analytical approach used in this study. It may be possible to use individual cases and population controls (matched by age, sex and region) as the units of analysis and traceback from all premises mentioned by cases and controls. Alternatively, rather than tracing food supply back from the venues, a trace-forward from a list of customers of an implicated supplier could be carried out. The relative resource implications of the traceback and trace-forward methodology would be influenced by the complexity of the food distribution network from the supplier to the retailer. In the period of interest, Supplier A supplied 96 companies, one of whom supplied over 600 further companies. This scale of food chain made a trace-forward approach impractical in the investigation timeframe with the available resources. An additional advantage of this method over a trace-forward approach is that it could be used with multiple suspected producers, or even where the source is initially unknown. Given that the ultimate producer for each food of interest would be traced, multiple hypotheses could be tested, although the addition of further food types would increase the workload involved.

We estimated that a substantial staff commitment was required from both PHE/PHW and local authorities to complete this study. This estimate could be regarded as a minimum, as we do not have the information to calculate the proportion of staff involved who completed the survey; EHOs may have been particularly under-represented. Although this study involved a sizeable allocation of resource, the outbreak investigation entailed no additional expenditure and the outbreak was an organisational priority.

### Limitations

The study design used has several potential limitations. Firstly, we know that only a proportion of *E. coli* O157 cases are reported and those that are reported may come with inaccurate or incomplete food history. It is therefore possible that there were unascertained cases linked to control venues. Secondly, cases will have had many different food exposures in the week before they became ill. It is possible that cases were exposed to the outbreak strain of *E. coli* elsewhere, not at the case venues. To reduce the impact of this potential limitation, we attempted a sensitivity analysis restricting the study to those premises associated with multiple cases. However, they were too few to estimate an effect. The result of both these potential limitations would be to reduce the observed strength of association; this would risk a type 1 error (missing a true association) but would not invalidate a positive association.

In this study, we restricted the inclusion criteria to venues serving salad leaves in an effort to ensure that case and control venues were comparable in that both used the relevant type of food. However, it is possible that control venues may have not served the same type of salad leaves as case venues and were therefore not in the same ‘source population’ as the case venues. After initiation of the study, information from other analytical studies indicated that mixed salad leaves were of greatest interest. However, it was not practical for the purposes of venue selection or data collection to restrict the study to venues serving mixed salad leaves. As we were not able to identify a specific product or batch, this study did not lead to any product recalls.

All venues meeting the case venue definition were included. If a case ate at more than one case venue in the 10 days before illness this may have led to underestimation of the association, as a venue which did not supply food contaminated with the pathogen would have been classed as a case venue. This was supported by the sensitivity analysis that we undertook, removing all venue pairs where the case also ate at another case venue linked to Supplier A. This removed three case pairs and increased the estimate of association.

In this study, we classified venues as being supplied by Supplier A if they received any salad leaves from this company. A number of venues received salad leaves from more than one supplier. In a number of cases, we therefore do not know definitively that the exact salad item eaten by cases at case venues was supplied by Supplier A. With regard to data collection, food business operators in the European Union have a legal obligation to ensure that in case of an investigation, traceability can be assured at all stages (EC Regulation 178/2002 [17]). It was our experience in this investigation that full traceability information (e.g. batch numbers) was frequently unavailable.

## Conclusion

We have described a novel application of the case–control study to investigate a food item which was widely distributed through the food chain to a variety of catering venues. While recognising the implicit assumptions, potential limitations and resource implications, we would recommend this analytical approach to investigators faced with a widely dispersed food-borne outbreak for which a common food item and specific supplier are implicated.
